# Progenitor Cell Line (hPheo1) Derived from a Human Pheochromocytoma Tumor

**DOI:** 10.1371/journal.pone.0065624

**Published:** 2013-06-13

**Authors:** Hans K. Ghayee, Vikash J. Bhagwandin, Victor Stastny, Arielle Click, Liang-Hao Ding, Dario Mizrachi, Ying S. Zou, Raj Chari, Wan L. Lam, Robert M. Bachoo, Alice L. Smith, Michael D. Story, Stan Sidhu, Bruce G. Robinson, Fiemu E. Nwariaku, Adi F. Gazdar, Richard J. Auchus, Jerry W. Shay

**Affiliations:** 1 Department of Internal Medicine, Division of Endocrinology, University of Texas Southwestern Medical Center, Dallas, Texas, United States of America; 2 Hooper Research Foundation, University of California San Francisco, San Francisco, California, United States of America; 3 Hamon Center for Therapeutic Oncology and Department of Pathology, University of Texas Southwestern Medical Center, Dallas, Texas, United States of America; 4 Department of Cell Biology, University of Texas Southwestern Medical Center, Dallas, Texas, United States of America; 5 Department of Radiation Oncology, University of Texas Southwestern Medical Center, Dallas, Texas, United States of America; 6 Chemical and Biomolecular Engineering, Cornell University, Ithaca, New York, United States of America; 7 Department of Pediatrics, University of Washington, Seattle, Washington, United States of America; 8 Department of Genetics, Harvard Medical School, Boston, Massachusetts, United States of America; 9 Department of Integrative Oncology, British Columbia Cancer Agency, Vancouver, British Columbia, Canada; 10 Department of Neurology, University of Texas Southwestern Medical Center, Dallas, Texas, United States of America; 11 Department of Surgery, Kolling Institute of Medical Research, St Leonards, New South Wales, Australia; 12 Department of Surgery, University of Texas Southwestern Medical Center, Dallas, Texas, United States of America; 13 Department of Medicine, Division of Metabolism, Diabetes, and Endocrinology, University of Michigan, Ann Arbor, Michigan, United States of America; 14 Center of Excellence in Genomic Medicine Research, King Abdulaziz University, Jeddah, Saudi Arabia; Baylor college of Medicine, United States of America

## Abstract

**Background:**

Pheochromocytomas are rare tumors generally arising in the medullary region of the adrenal gland. These tumors release excessive epinephrine and norepinephrine resulting in hypertension and cardiovascular crises for which surgery is the only definitive treatment. Molecular mechanisms that control tumor development and hormone production are poorly understood, and progress has been hampered by the lack of human cellular model systems. To study pheochromocytomas, we developed a stable progenitor pheochromocytoma cell line derived from a primary human tumor.

**Methods:**

After IRB approval and written informed consent, human pheochromocytoma tissue was excised, minced, dispersed enzymatically, and cultured in vitro. Primary pheochromocytoma cells were infected with a lentivirus vector carrying the catalytic subunit of human telomerase reverse transcriptase (hTERT). The hTERT immortalized cells (hPheo1) have been passaged >300 population doublings. The resulting cell line was characterized morphologically, biochemically and for expression of neuroendocrine properties. The expression of marker enzymes and proteins was assessed by immunofluorescence staining and immunoblotting. Telomerase activity was determined by using the telomeric repeat amplification protocol (TRAP) assay.

**Results:**

We have established a human pheochromocytoma precursor cell line that expresses the neuroendocrine marker, chromogranin A, when differentiated in the presence of bone morphogenic protein 4 (BMP4), nerve growth factor (NGF), and dexamethasone. Phenylethanolamine N-methyltransferase (PNMT) expression is also detected with this differentiation regimen. CD-56 (also known as NCAM, neural cell adhesion molecule) is expressed in these cells, but CD31 (also known as PECAM-1, a marker of endothelial cells) is negative.

**Conclusions:**

We have maintained hTERT-immortalized progenitor cells derived from a pheochromocytoma (hPheo1) in culture for over 300 population doublings. This progenitor human cell line is normal diploid except for a deletion in the p16 region and has inducible neuroendocrine biomarkers. These cells should be a valuable reagent for studying mechanisms of tumor development and for testing novel therapeutic approaches.

## Introduction

Pheochromocytomas are catecholamine-producing tumors, which arise from chromaffin cells in the adrenal medulla. Pheochromocytomas and paragangliomas (extra-adrenal pheochromocytomas) usually produce catecholamines. The incidence of pheochromocytoma in the USA is 1–2 per million [1]. Patients with pheochromocytomas demonstrate variable clinical presentations including spells with paroxysmal hypertension and palpitations, and symptoms may mimic pre-eclampsia in pregnant women [2]. If untreated, pheochromocytomas can lead to cardiovascular collapse or death due to excess catecholamine production. Surgical excision of pheochromocytomas represent the only curative therapy, although cytotoxic chemotherapy, high dose [^131^I]-metaiodobenzylguanidine (MIBG) [3], and tyrosine kinase inhibitors [4] all show some activity in metastatic disease. The basis of some of these treatment options is based on the discovery of a number of genes associated with pheochromocytomas, including neurofibromin 1 (*NFI*), von Hippel-Lindau (*VHL*), transmembrane 127 (*TMEM127*), proto-oncogene (*RET*), and MYC associated factor X (*MAX*) [5], [6]. Genes associated with extra-adrenal pheochromocytomas (paragangliomas) include four nuclear genes that encode subunits of the mitochondrial enzyme succinate dehydrogenase (*SDHA*, *SDHB*, *SDHC*, *SDHD*), which catalyzes the conversion of succinate to fumarate in the Krebs Cycle [7] in addition to having an association with the hypoxia pathway. *HIF2* is another gene associated with the hypoxia pathway and paragangliomas [8]. Despite some advancement in the genetics associated with pheochromocytomas, the exact mechanisms of how these tumors form and how the specific gain or loss of function of genes involved in the pathogenesis of this disease are still largely unknown. Fortunately, the recurrence and metastasis of pheochromocytomas are rare; however, metastases are associated with a 5 year survival of <40% [9] [10].

While molecular mechanisms that control pheochromocytoma development remain poorly understood, progress is further hampered by the lack of suitable model systems, limited to the PC-12 rat pheochromocytoma cell line [11], mouse pheochromocytoma (MPC) cell line [12], and recently developed mouse MPC derivative known as MTT [13]. Attempts at developing human pheochromocytoma cell lines have not been successful, most likely due to the limited life spans of normal human cells in culture [14]. Establishing cell lines from normal tissues and benign tumors is challenging, since telomere shortening and lack of cell cycle augmentation derived from the characteristic of the transformed phenotype thwart long-term propagation. Previous reports [15], [16] have shown that by introducing human telomerase reverse transcriptase (hTERT) into human cells, with or without introduction of cyclin dependent kinase 4 (CDK4), can lead to immortalization of cells with minimal alteration of cell phenotype. To date, this method has been applied to non-malignant cells including human bronchial, mammary, retinal, colonic, skin epithelial cells, skeletal muscle cells, vascular endothelial cells, and fibroblasts [15]–[18]. We applied this technology in an attempt to immortalize endocrine tumors of low or unknown malignant potential and to develop a cell line from a human pheochromocytoma, by stably introducing hTERT alone. The result is that we have developed a unique neuroendocrine progenitor cell line derived from a human pheochromocytoma tumor that should have utility in dissecting molecular pathways that influence growth and differentiation leading to pheochromocytoma.

## Methods

### Case

A 39 year-old woman presented for work-up of recurrent nephrolithiasis and was incidentally found to have a 4 cm left adrenal mass. She did not have hypercalcemia or any family history of pheochromocytoma, hyperparathyroidism, or thyroid cancer. Work-up of this mass revealed elevated 24 hr urine normetanephrine of 1120 µg/24 hrs (<900) and metanephrine of 973 µg/24 hrs (<400). Norepinephrine in the 24 hr urine collection was 37 µg/24 hrs (15–80), epinephrine was 12 µg/24 hrs (0–20), and dopamine was 200 µg/24 hrs (65–400). Her plasma normetanephrine of 3.09 nmol/L (<0.90) and metanephrine of 0.86 nmol/L (<0.50) were also elevated. She did not have cortisol and aldosterone hypersecretion. In retrospect, she did report having episodic symptoms of tachycardia and nervousness. She was referred for a left adrenalectomy. Pathology confirmed that this tissue was a pheochromocytoma.

### Isolation of Cells Derived from a Human Pheochromocytoma

Tissue from this woman’s pheochromocytoma was minced into small pieces and incubated with collagenase type 4 at 2.5 mg/ml (Worthington # 46K8986) along with deoxyribonuclease I at 0.05 mg/ml (Worthington # S7M9938F) [19], [20], and mixed with 12 ml Hank’s Buffer Salt Solution (HBSS) for 3 hours at 37C. The digested tissue was dispersed into a single cell suspension by pipetting and centrifuged at 1000 rpm for 5 min. The supernatant was aspirated, and the cell pellet was resuspended and maintained as nonadherent spheroids in a chemically defined serum-free DMEM/F-12 (Cellgro), consisting of human recombinant epidermal growth factor (20 ng/ml; Sigma), basic fibroblast growth factor (20 ng/ml; Upstate), B27 supplement (1×; Invitrogen), insulin-transferrin-selenium-X (1×; Invitrogen), and penicillin-streptomycin (100 units/ml and 100 µg/ml; HyClone) [21]. In this medium, fibroblasts remained attached to the polystyrene plate (standard tissue culture coating), while the neuroendocrine cells remained in suspension as spheroids. After 2 weekly passages, the medium was switched to ACL4 medium [22] with 10% fetal bovine serum, where cells settled on polystyrene T-75 flask (standard tissue culture coating). Cells were passaged with approximately two population doublings occurring per week.

### Lentiviral Production

HEK293FT cells were plated at a density of 10e^6^ cells per 10 cm dish 24 hours prior to transfection. The cells were transfected with the hTERT lentiviral vector along with packaging vectors, pMD2G, and psPAX2 using the manufacturer’s suggested protocol for FuGENE6. The transfection medium was removed the following morning and replaced with standard ACL4 growth medium. Supernatants from these cells were collected 24 hours after recovery from transfection. The supernatants were filtered using a 0.45 µM syringe filter, combined with 4 µg/ml of polybrene, and added to the cultured primary pheochromocytoma cells.

### hTERT Immortalization of Pheochromocytoma Cells

Primary pheochromocytoma cells were infected with lentivirus-hTERT containing a blasticidin resistance gene. Pheochromocytoma cells were selected at six weeks post infection (in order to allow enough cells to grow for selection) with blasticidin (2 mg/ml) for 10 days. The telomeric repeat amplification protocol (TRAP) assay was used to determine whether the blasticidin resistant cells were expressing the hTERT catalytic subunit using the TRAPeze kit (Chemicon, Temecula CA) according to manufacturer’s instructions. The telomerase products (6-bp ladder) and the 36-bp internal control (ITAS) bands were quantified using the AlphaImager 2000 software (Alpha Innotech Corporation, San Leandro CA). Relative telomerase activity (RTA) was calculated as the intensity ratio of the TRAP ladder to that of the ITAS band (relative intensity of each sample was normalized to that of the positive control). See Figure S1.

### Characterization of Immortalized Pheochromocytoma Cell Line

#### Measurement of catecholamines

Samples were prepared by sonicating approximately 1 million cells in 15 ml conical tubes with 200 µl of 2% formic acid in water. Methanol was added to precipitate proteins, and the samples were centrifuged. Samples were analyzed by an MDS Sciex API 5000, equipped with turbo ion spray probe coupled to a Shimadzu LC system. Separation of epinephrine (E), norepinephrine (NE), and normetanephrine (NMN) was achieved with a Phenomynex LUNA Cyano analytical column (15 cmX4.6 mm; 5 µm particle size) and with a Phenomynex Cyano guard column. Mobile phase was acetonitrile-water (60:40 by volume) containing 1.5 mmol/L ammonium acetate and 0.6 g/L formic acid. Flow rate was 0.5 ml/min. Ionization of E, NE, and NMN was performed in positive mode with purified nitrogen as the curtain gas with zero air for Gas 1/Gas 2. Mass Spectrometer settings were temperature = 500C, dwell time = 200 ms, declutering potential = 26, entrance potential = 10, collision energy = 29, collision exit potential = 6, Q1 resolution = unit, Q3 resolution = unit, collision gas = 8, curtain gas = 20, ionsource gas 1 (GS1) = 25, ionsource gas 2 (GS2) = 25, and ionspray voltage = 5500. 20 µl of the sample was injected onto the column. Acquisition was achieved in multiple-reaction monitoring mode (MRM). Transitions of the precursor ions to product ions were m/z 152→107 for NE, m/z 184→107 for E, and m/z 184→166 for NMN. Total chromatographic run time was 6 minutes. Quantitative analysis was by peak height.

#### Immunoblotting

Immunoblot for tyrosine hydroxylase (TH), dihydroxyphenylalanine decarboxylase (DOPADC), dopamine β-hydroxylase (DBH), and catecholamine-O-methyltransferase (COMT) was performed using antibodies from Santa Cruz Biotechnology (TH, #14007; DOPADC, #46909; DBH, #33914; and COMT, #25844). Chromogranin A antibody was purchased from Millipore. Protein extraction was achieved using lysis buffer (Cell Signaling, #9803) containing a full spectrum of protease inhibitors. Test samples were loaded using 25 µg protein/well. Proteins were resolved with 10% SDS-PAGE and transferred to PVDF membranes (Immobilon-P, Millipore) over 1 hour at 86 volts. The membrane was washed with TTBS (Tris•HCl buffer pH 7.5, 0.5% Tween-20) twice for 5 min, then blocked by shaking with 5% fat-free milk in TTBS for one hour at room temperature. The blots were then probed with primary antibody at 1∶1000 in 5% milk/TTBS overnight at 4C. After washing with TTBS, the secondary antibody (either rabbit anti-goat IgG-HRP or goat anti-rabbit IgG-HRP from Bio-Rad, Richmond, CA, 1∶5000 in 5% milk/TTBS) was added for 1 h at room temperature. The membrane was washed with TTBS and developed using the Thermo Scientific Westfemto kit (PI34095).

#### Immunofluorescence staining

Cells were plated at 50% density in 24 well plates on sterile glass. They were later fixed in 3.7% formalin in PBS. After several washes in PBS, the cells on glass coverslips were blocked in normal goat serum, washed again, and subsequently primary CD56 antibody or CD31 antibody (Cell Signaling) was added for overnight incubation. Cells were then washed and respective secondary antibodies conjugated with Alexa Fluor were added for 1 hr and then counterstained with DAPI. After a final wash and slide mounting preparation, images were taken at 40X with 350 ms exposure time for rhodamine and 50 ms exposure time for DAPI.

#### Conventional chromosomal studies

Chromosome analysis was performed on metaphase cells derived from a short-term culture of cell lines according to the standard procedure [23]. Slides were G-banded for karyotyping analyses.

#### SNP array profiling and feature extraction

DNA (500 ng) from the pheochromocytoma cell line was profiled using the Genome-Wide Human SNP Array 6.0 platform. GeneChip Command Console Software was used to generate feature extracted intensity files, which were processed using the Birdseed V2 algorithm in the Genotyping Console to create genotype call files.

#### DNA copy number, allelic status, and SNP analyses

Total copy number and allelic status analyses were determined using Partek Genomics Suite (PGS). Specifically, for total copy number analysis, the “Copy Number Analysis” workflow was used whereby the reference copy number for each sample was determined using a pool of 72 normal samples from the HapMap collection. Regions of copy number gain and loss were then statistically detected using the Genomic Segmentation method within PGS using default parameters, except for the “Number of Markers” parameter, which was changed to 50. Single nucleotide polymorphism (SNP) array analysis was performed through the BC Cancer Research Centre Array Laboratory.

#### Molecular fingerprinting

Genomic DNA was extracted from cell pellets using the QIAamp DNA Blood Mini Kit (Qiagen), concentrated using a Speed-Vac, and diluted to 20 ng/µl. DNA (20 ng) was genotyped via the Powerplex panel of 9 highly informative microsatellite markers (Promega, San Luis Obispo, CA) [24]. The resultant haplotype of the pheochromocytoma cell line was compared to those of other common cell lines in use in our laboratories and those of the American Type Culture Collection (ATCC, Manassas, VA).

#### Microarray sample labeling, hybridization and data processing

Total RNA was extracted from tissues and cells using Qiagen RNeasy Mini Kit according to manufacturer’s instruction. Illumina Whole Genome HumanWG6 v3 Expression BeadChip was used in this study. Each RNA sample with 0.5 µg of total RNA was amplified using the Illumina TotalPrep RNA amplification kit with biotin UTP (Enzo) labeling. The Illumina TotalPrep RNA amplification kit uses T7 oligo(dT) primer to generate single-stranded cDNA followed by a second strand synthesis to generate double-stranded cDNA, which is then column purified. In vitro transcription was done to synthesize biotin labeled cRNA using T7 RNA polymerase. The cRNA was then column purified. The cRNA was then checked for size and yield using the Bio-Rad Experion system. 1.5 µg of cRNA was hybridized for each array using standard Illumina protocols with streptavidin-Cy3 (Amersham) being used for detection. Slides were scanned on an Illumina Beadstation. Summarized expression values for probe sets were generated using BeadStudio 3.1 (Illumina, Inc). The data were background subtracted and quantile-quantile normalized across samples using MBCB algorithm [25].

#### Clustering analysis

Hierarchical clustering analysis and dendogram generation were performed using Partek Genomics Suite. Normalized gene expression values were used to cluster samples. Euclidean Distance was used as dissimilarity matrix, and Complete Linkage was used as clustering method.

#### Cell differentiation

Differentiation was induced by adding 20 ng/ml BMP4 (R&D Systems), 20 ng/ml nerve growth factor (NGF, Sigma-Aldrich), and 10 µM dexamethasone (Sigma-Aldrich). The basal media used was composed of DMEM/F-12 containing 20 ng/ml basic fibroblast growth factor (bFGF, Sigma-Aldrich), 20 ng/ml epidermal growth factor (EGF, Sigma-Aldrich), 1% insulin transferring selenium (ITS) (Gibco), and 10 ng/ml leukemia inhibitory factor (LIF, Sigma-Aldrich). Differentiation protocol was followed as described [26].

#### shRNA knockdown of SDHB, RET, and COMT

shRNA was used for knockdown of specific genes including *SDHB, RET,* and *COMT.* Five million 293FT cells were plated per 10 cm plate in Media X. The medium was changed the next day. Five micrograms of plasmid DNA was diluted to 2 µg pMD2G and 3 µg psPAX2 plasmids into 250 µl medium, which was vortexed and spun down. Thirty microliters of polyjet was diluted into 250 µl medium, which was also vortexed and spun down. Diluted polyjet was added to diluted plasmid DNA, vortexed, spun down, and incubated for 20 minutes. Five hundred microliters of the transfection complex was added to the 293FT cells. Twenty-four hours after transfection, the medium was changed. Forty-eight and seventy-two hours after transfection, virus was collected and filtered with 0.45 µM filter. Using 4 mL virus mixed with 6.25 µl polybrene, the hPheo1 cells were infected. The medium was changed again eight hours later. Since the shRNA vectors contained green fluorescence protein (GFP) and a puromycin selectable marker, GFP expression was assessed two days later as well as a selection for puromycin resistance (2 mg/ml).

#### qPCR

RNA was extracted from cell pellets using Qiagen RNeasy kit (cat# 74104). The Bio-Rad iScript (cat# 170–8890) reverse transcriptase was used to synthesize cDNA. The mastermix used for the qPCR was Bio-Rad SsoFast probes mix (cat# 172–5230), and the probes were from the Roche universal probe library (cat# 04683633001) and Invitrogen taqman probes. The qPCR was run on a Roche 480 light cycler.

## Results

### Establishment and Properties of hPheo1 Cells

#### hPheo1 immortalized cell line derived from human pheochromocytoma

Lentivirus-hTERT infected cells were selected with blasticidin (for 10 days) and then passaged for >300 population doublings, establishing the line. These cells demonstrate high telomerase activity compared to the negative control, primary culture pheochromocytoma cells, using the TRAP assay (Figure S1). Growth of this cell line in ACL4 medium with 10% fetal bovine serum (FBS) averaged 2 population doublings (PD) per week initially, then growth increased to 6 PD/week with cells growing in 5% FBS. Cultures could be established from previously cryopreserved cells (in fetal bovine serum with 10% dimethylsulfoxide) without difficulty. Molecular analysis of the immortalized cell line by DNA fingerprinting (Figure S2) did not match any other cell line in our database or in that of the ATCC. These cells have now been maintained in culture for ∼5 years, and this immortalized line derived from a human pheochromocytoma is referred to as hPheo1.

### Characterization of hPheo1 Cells

#### hPheo1 have loss of p16 gene and can be re-differentiated but do not make catecholamines

**Figure 1:**
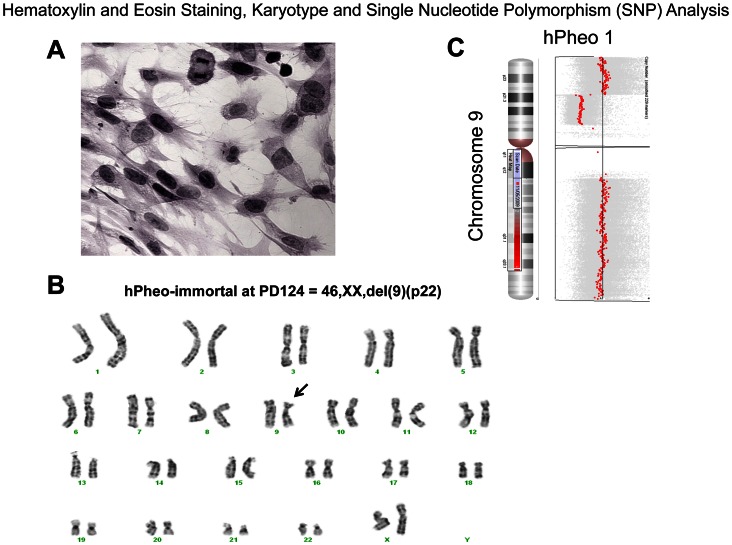
Characterization of hPheo1 cells. A. Hematoxylin and eosin staining shows plump polygonal cells with eosinophilic cytoplasm. B. Karyotypic analysis shows a female diploid pattern with a single cytogenetic abnormality, del9p22 on one chromosome, near its telomeric end (arrow). C. SNP analysis confirms the deletion of 9p, which encompasses the *CDKN2A* (p16 gene).

**Figure 2:**
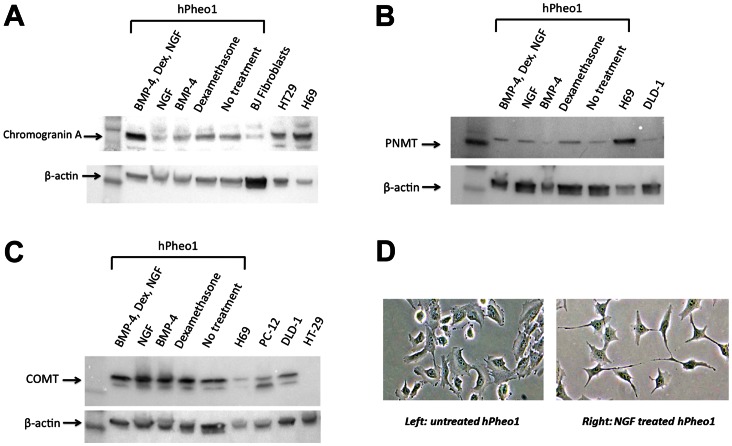
Differentiation of hPheo1 cells. A. Western blot for chromogranin A shows expression more prominent after differentiation treatment. B. Western blot for PNMT shows expression more prominent after differentiation treatment. C. Presence of COMT in hPheo1 cells is seen, whether treated with differentiating factors or not. D. Neurite formation in hPheo1 cells occurs after treatment with Nerve Growth Factor (NGF).

**Figure 3:**
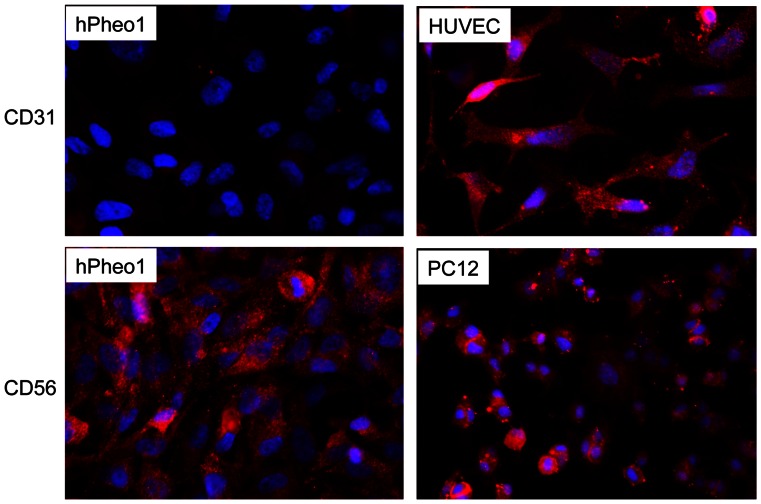
Immunofluorescence reveals CD56 is positive in hPheo1 and PC-12 cells. **CD31 is negative in hPheo1 but positive in HUVEC cells.**

**Figure 4:**
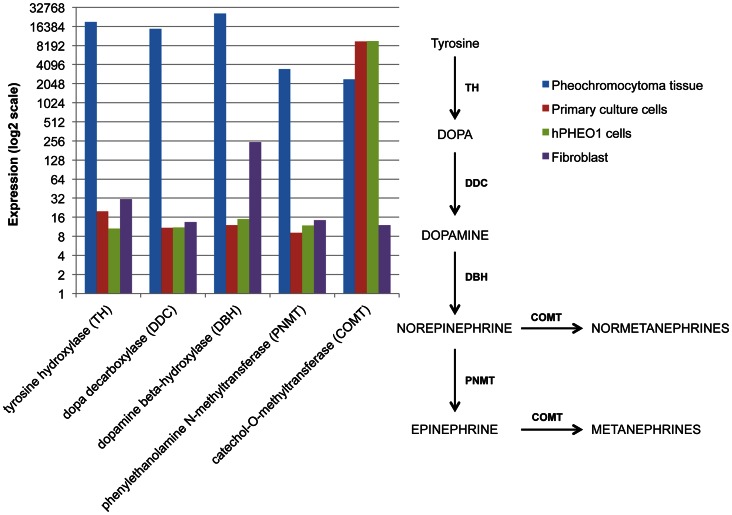
RNA expression of catecholamine enzyme synthesis. Enzyme expression for catecholamine synthesis is down-regulated in hPheo1 cells, however COMT expression is increased.

The cytological appearance of the cells are shown in Figure 1A. hPheo1 cells have high level expression of chromogranin A after induction with BMP4, NGF, and dexamethasone (Figure 2A). In addition, hPheo1 cells express PNMT after induction with BMP4, NGF, and dexamethasone (Figure 2B). This indicates that hPheo1 cells have neuroendocrine properties. Karyotype and SNP analyses demonstrated a small deletion of chromosome 9p on one chromosome, encompassing the *CDKN2A* gene encoding the p16 protein (Figure 1B and 1C). No other cytogenetic change was noted. The SNP analysis of a portion of the tumor tissue did not show a microdeletion on 9p, at least at the gross level. Instead, tumor tissue shows the cytogenetic changes −1p, +3p, −3q, −4q, segmental losses on 11p and 11q, and −17. Immunohistochemistry (IHC) for CD56 (NCAM, neural cell adhesion molecules) is positive for hPheo1 cells and PC-12 cells as shown in Figure 3. In contrast, IHC for CD31 (endothelial cell marker) is negative for hPheo1 cells but positive for the human umbilical vein endothelial cell line, HUVEC. This demonstrates that hPheo1 cells are not endothelial cells. From RNA expression (Figure 4), enzymes associated with the catecholamine pathway are down-regulated, however COMT expression is increased. Figure 2C confirms COMT expression by western blot. Also, confirmed by western blot was lack of expression of TH, DOPADC, DBH (data not shown).

### Molecular Fingerprinting

#### hPheo1 is a new human progenitor cell line

As shown in Figure S2, genomic analysis of hPheo1 cells compared with the Powerplex panel of 9 highly informative microsatellite markers, demonstrate that hPheo1 cells are a new and unique cell line, which has not been reported before.

### Microarray Gene Expression

#### hPheo1 cells have high COMT expression

Figure S3 shows clustering analysis of gene expression profiles of human skin fibroblasts, hPheo1 cells, pheochromocytoma tumor tissue from which the hPheo1 cells were derived, and three normal adrenal medullary tissues. The gene expression profiles between hPheo1 cells, tumor tissue, and normal adrenal medulla show distinctly different expressions from fibroblasts, indicating that hPheo1 cells are not fibroblasts. Figure 4 shows the RNA expression of enzymes for catecholamine synthesis. All of the enzymes are up regulated in the tumor tissue (blue bars), but not in hPheo1 cells or primary culture cells, except for COMT. When comparing COMT expressions between the groups, COMT expression is 8-fold higher in hPheo1 and in primary cultures compared with the tumor tissue. COMT is known to normally degrade catecholamines.

### shRNA was used to Knockdown SDHB, RET and COMT as an Attempt to Re-differentiate hPheo1 Cells

#### qPCR showed no evidence of re-differentiation of hPheo1 cells after gene knockdowns

Figure S4 shows qPCR results indicating evidence of reduced expression of *SDHB, RET,* and *COMT* in hPheo1 cells after respective shRNA knockdowns. Figure S5 shows no up-regulation in the differentiation markers of TH, DBH or chromogranin A after stable shRNA knockdown of *SDHB, RET,* and *COMT.* With *RET* knockdown, there was a minimal increase in DOPADC, yet the expression of other components for catecholamine synthesis were either unchanged or low. On the other hand, with *SDHB, RET,* and *COMT* knockdowns, DBH expression was more reduced compared to other differentiation markers.

## Discussion

It has been difficult to grow human pheochromocytoma cells in culture because they derive from low-grade benign tumors that are often telomerase negative. Therefore, we adopted an approach to immortalize pheochromocytoma cells utilizing hTERT. Previous reports [15], [16] describing immortalization of cells include infecting cells with virus expressing CDK4 in order to overcome p16-induced premature senescence, so that the cell cycle continues to proceed. In contrast, it was not necessary for us to add CDK4, as hPheo1 cells were found to have a deletion in *CDKN2A* (p16 gene) with no other large genetic changes noted on karyotype and SNP analysis. It is unclear whether mutations in p16 are important for oncogenesis of pheochromocytoma. An earlier study using semi-quantitative PCR indicated that deletion of the p16 gene may not be common in pheochromocytoma [27], so this loss could have occurred during the establishment of the cells in culture. In contrast, a recent report utilizing data from tissue microarray, quantitative real-time PCR, and methylation-specific PCR noted an overall frequency of genetic alterations involving the p16 gene in over 60% of pheochromocytomas [28].

The tumor tissue from which hPheo1 cells are derived did not grossly have a p16 gene deletion, however, it did show additional cytogenetic alterations not found in the hPheo1 line. Of note, alterations of −1p, −3q, along with losses of 11p and 11q, have been previously reported in pheochromocytoma SNP analysis [29]. Fingerprinting analysis between the cell line and the tumor tissue confirm that both cell populations derive from the same patient. We speculate that the discrepancy in the SNP data between the cell line and tumor tissue reflects that the line arose from a subclonal population of progenitor tumor cells, which acquired the necessary mutation (loss of p16) in order to survive but did not contain some of the other molecular changes in the bulk of the tumor. Besides absence of p16, we believe hPheo1 cells are a stable line since these cells retain high telomerase activity for over 300 population doublings. In contrast, the primary pheochromocytoma cell cultures (without hTERT) ceased to grow after eight passages (∼15 PDs). Microarray expression data were examined to compare common contaminant cells in tissue culture such as fibroblasts to hPheo1 cells, shown in Figure S3. The hierarchical clustering analysis clearly indicated that the gene expression profile of hPheo1 cells belong to the same group as tumor and the normal adrenal medulla, but very different from fibroblasts. Data from immunofluorescence staining also demonstrate that hPheo1 cells are not endothelial cells. Comparison analysis of the gene expression analysis between hPheo1 cells and the tumor tissue from which they derive also show many differences, yet several genes are highly expressed in both hPheo1 cells and pheochromocytoma tumor tissue, including transforming growth factor-beta receptor III (TGFBR3), involved in differentiation between benign and malignant pheochromocytomas [30]; LOC399942; tubulin alpha-2 chain transcript variant 5, involved with overexpression in neuroblastoma [31]; and breast cancer anti-estrogen resistance 1 (BCAR1), related to the accelerated differentiation of PC-12 cells in response to nerve growth factor (NGF) [32]. With NGF treatment, neurites develop in hPheo1 cells (Figure 2D) and properties of differentiation become more apparent. Since the hPheo1 cells have fewer cytogenetic changes than most cells in the primary tumor, it is possible that the line derives from a premalignant clone that had yet to reach terminal telomere shortening and was rescued by the addition of hTERT in vitro. This explanation is consistent with the model that premalignant cells must reach critical telomere shortening, followed by genomic instability, and then reactivation of telomerase to allow continued cell proliferation. Our data are consistent with this interpretation and suggest that hPheo1, a progenitor line, may be a useful cell reagent for additional cancer progression studies.

All of the genes associated with catecholamine synthesis are highly expressed in the tumor tissue, but most are down-regulated in hPheo1 cells (Figure 4) related to dedifferentiation or down-regulation of some enzymes due to adaptation to monolayer culture. In contrast, expression of COMT, which is involved in breakdown of catecholamines, is 8-fold higher in hPheo1 cells compared to the primary tumor as noted in RNA expression. Since COMT is important for the degradation of catecholamines, shRNA was used to knockdown COMT; however, this did not induce the up-regulation of TH, DOPADC, or DBH for catecholamine production. It appears that the inherent up-regulation of COMT did not necessarily metabolize any catecholamines that might be produced. In addition, shRNA was used to knockdown other genes associated with pheochromocytoma pathogenesis such as *SDHB* (Figure S5), yet markers for re-differentiation were not up-regulated either. A possible explanation is that the genes associated with pheochromocytoma pathogenesis have been implicated more so with tumor growth than differentiation. However, the expression of chromogranin A and PNMT being more robustly inducible with BMP4, NGF, and dexamethasone does provide additional evidence that these cells are neuroendocrine in nature. Independent of hormone production, these cells should be useful for studying signaling pathways controlling growth and metastasis.

The rat and mouse pheochromocytoma cell lines are the most commonly used cell reagents to study the cell biology of pheochromocytoma, yet they might not utilize the same signaling pathways for growth and differentiation as in human tumors. Here, we describe the generation and characterization of a progenitor cell line derived from a human pheochromocytoma, hPheo1. The molecular alterations in these cells might provide clues to the pathways that are essential for catecholamine synthesis and explain why some of these tumors remain biochemically silent. Further study of hPheo1 cells will increase our understanding of the fundamental mechanisms involved in oncogenesis and tumor progression, which might facilitate the development of new treatment targets for pheochromocytoma.

## Supporting Information

Figure S1 **Telomeric Repeat Amplification Protocol (TRAP) assay.** Like the positive control Hela cells, immortalized hPheo1 cells show evidence of telomerase activity.
(TIF)

Figure S2 **Molecular fingerprint for hPheo1.** First column shows patient is a normal female XX. CSF1PO, D13S317, D16S539, D5S818, D7S820, THO1, TPOX, and vWA are microsatellite markers for fingerprinting analysis. hPheo1 is a new and unique cell line.
(TIFF)

Figure S3 **Microarray comparing expression profiles of fibroblasts with hPheo1 cell line, pheochromocytoma tumor tissue, and normal adrenal medullary tissues.** Expression profiles between hPheo1 cells, tumor tissue, and normal adrenal medulla show distinctly different expressions from fibroblasts.
(TIFF)

Figure S4 **Fold change expression from shRNA knockdown.** There is decreased expression of *SDHB, RET,* and *COMT* after shRNA knockdown.
(TIFF)

Figure S5 **hPheo1 cells do not show re-differentiation with shRNA knockdown of** ***SDHB*** **,** ***RET*** **, and** ***COMT*** **when assessing changes in tyrosine hydroxylase, dopa decarboxylase, dopamine beta-hydroxylase, and chromogranin A.**
(TIF)
