# Supplementary figures and images for: Progenitor Cell Line (hPheo1) Derived from a Human Pheochromocytoma Tumor
Source: PLoS One. 2013 Jun 13;8(6):e65624. doi: 10.1371/journal.pone.0065624 (PMC3681983; doi:10.1371/journal.pone.0065624)

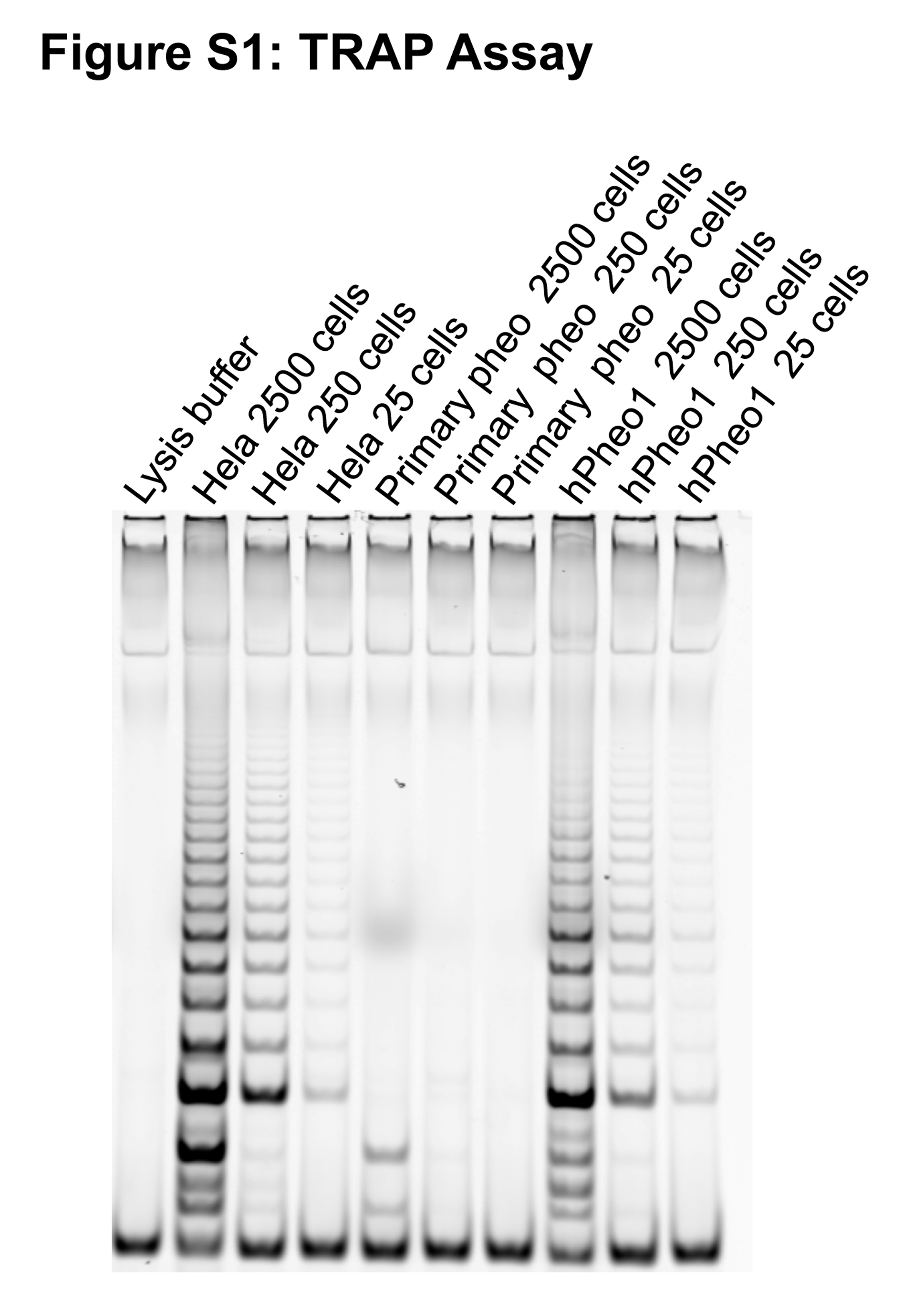

Supplement: Figure S1 — Telomeric Repeat Amplification Protocol (TRAP) assay. Like the positive control Hela cells, immortalized hPheo1 cells show evidence of telomerase activity. (TIF) [file pone.0065624.s001.tif]

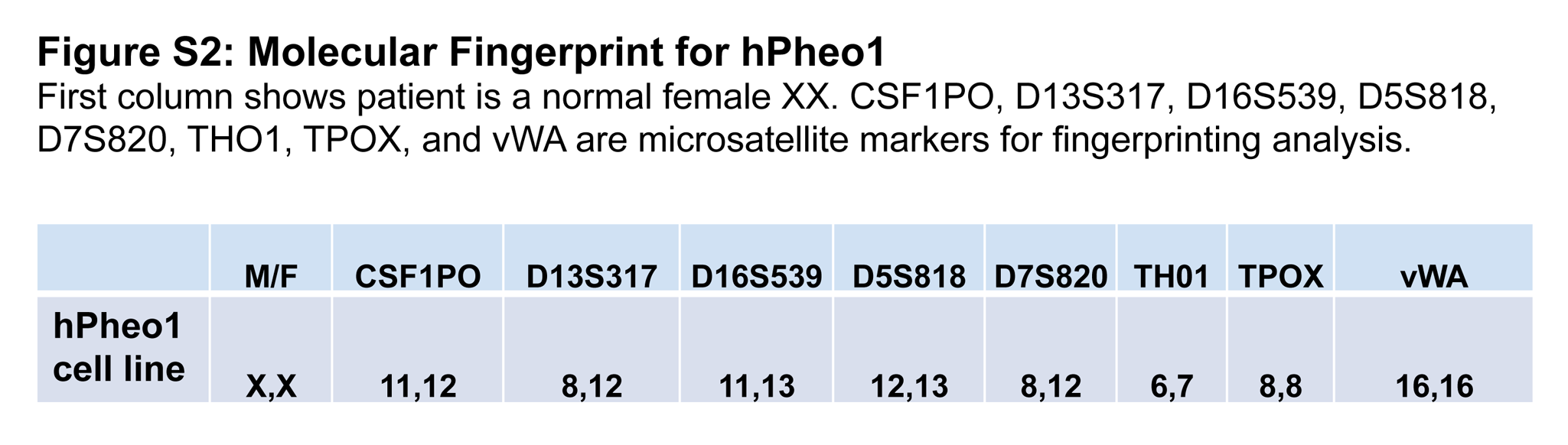

Supplement: Figure S2 — Molecular fingerprint for hPheo1. First column shows patient is a normal female XX. CSF1PO, D13S317, D16S539, D5S818, D7S820, THO1, TPOX, and vWA are microsatellite markers for fingerprinting analysis. hPheo1 is a new and unique cell line. (TIFF) [file pone.0065624.s002.tif]

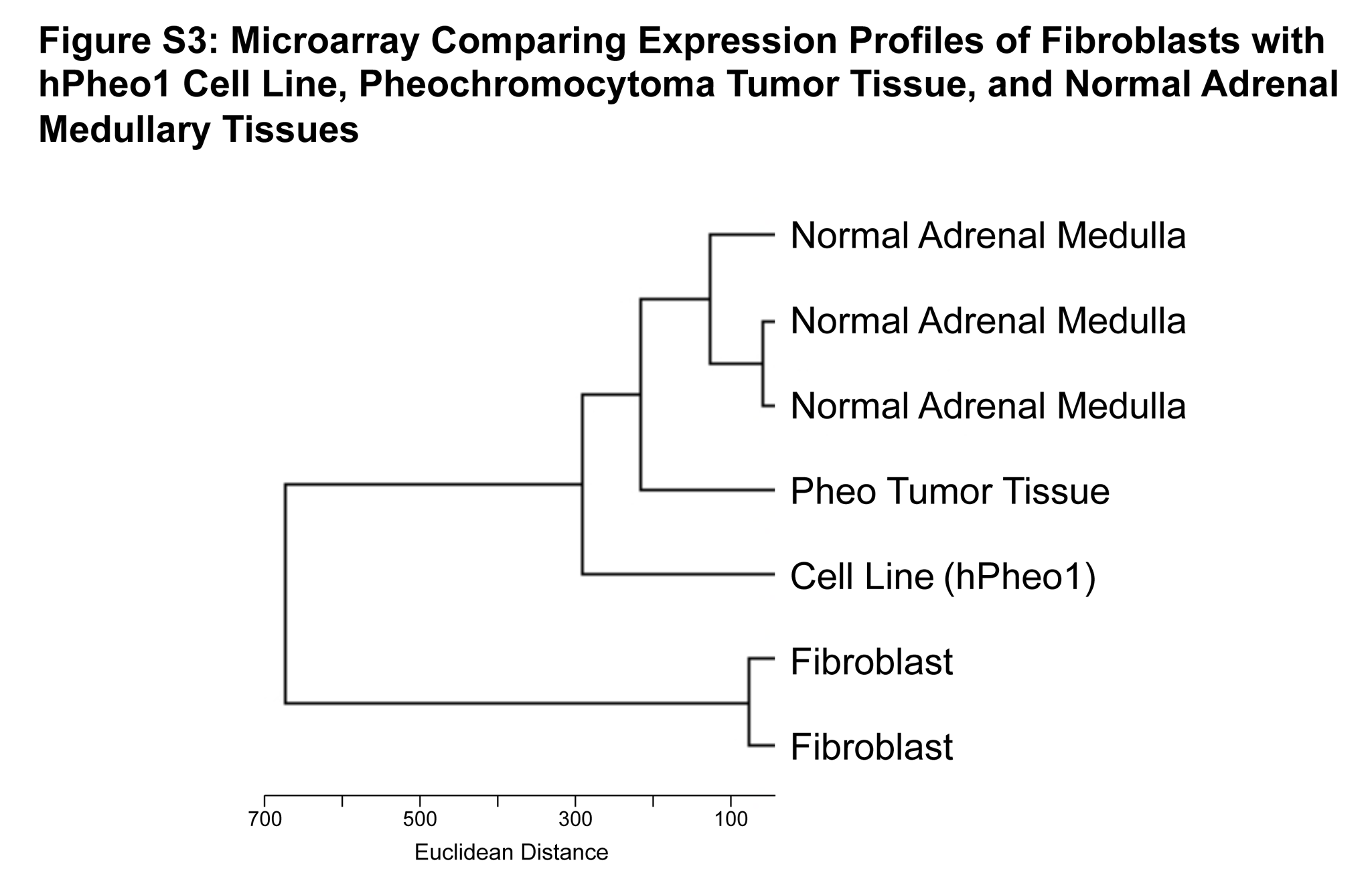

Supplement: Figure S3 — Microarray comparing expression profiles of fibroblasts with hPheo1 cell line, pheochromocytoma tumor tissue, and normal adrenal medullary tissues. Expression profiles between hPheo1 cells, tumor tissue, and normal adrenal medulla show distinctly different expressions from fibroblasts. (TIFF) [file pone.0065624.s003.tiff]

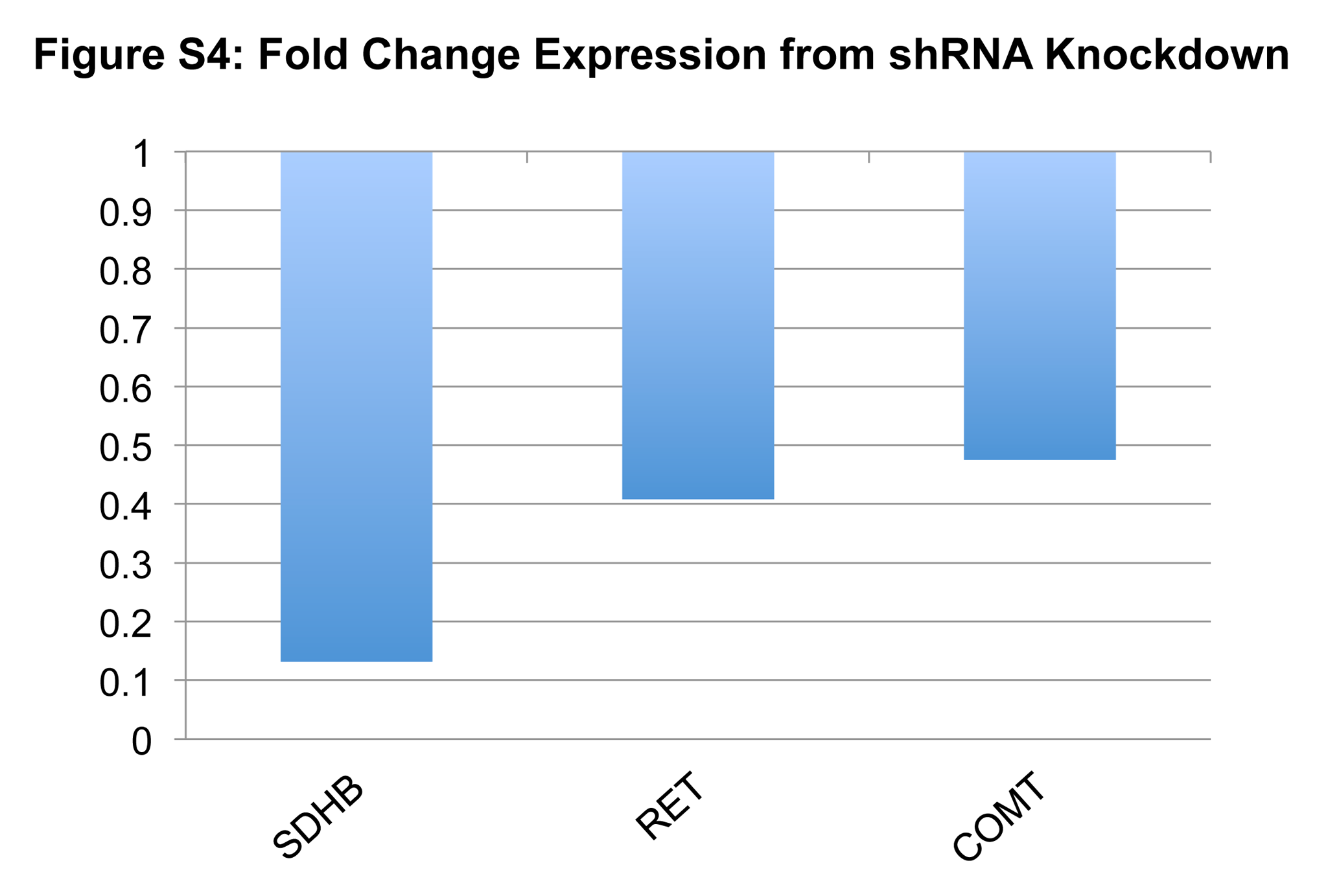

Supplement: Figure S4 — Fold change expression from shRNA knockdown. There is decreased expression of SDHB, RET, and COMT after shRNA knockdown. (TIFF) [file pone.0065624.s004.tiff]

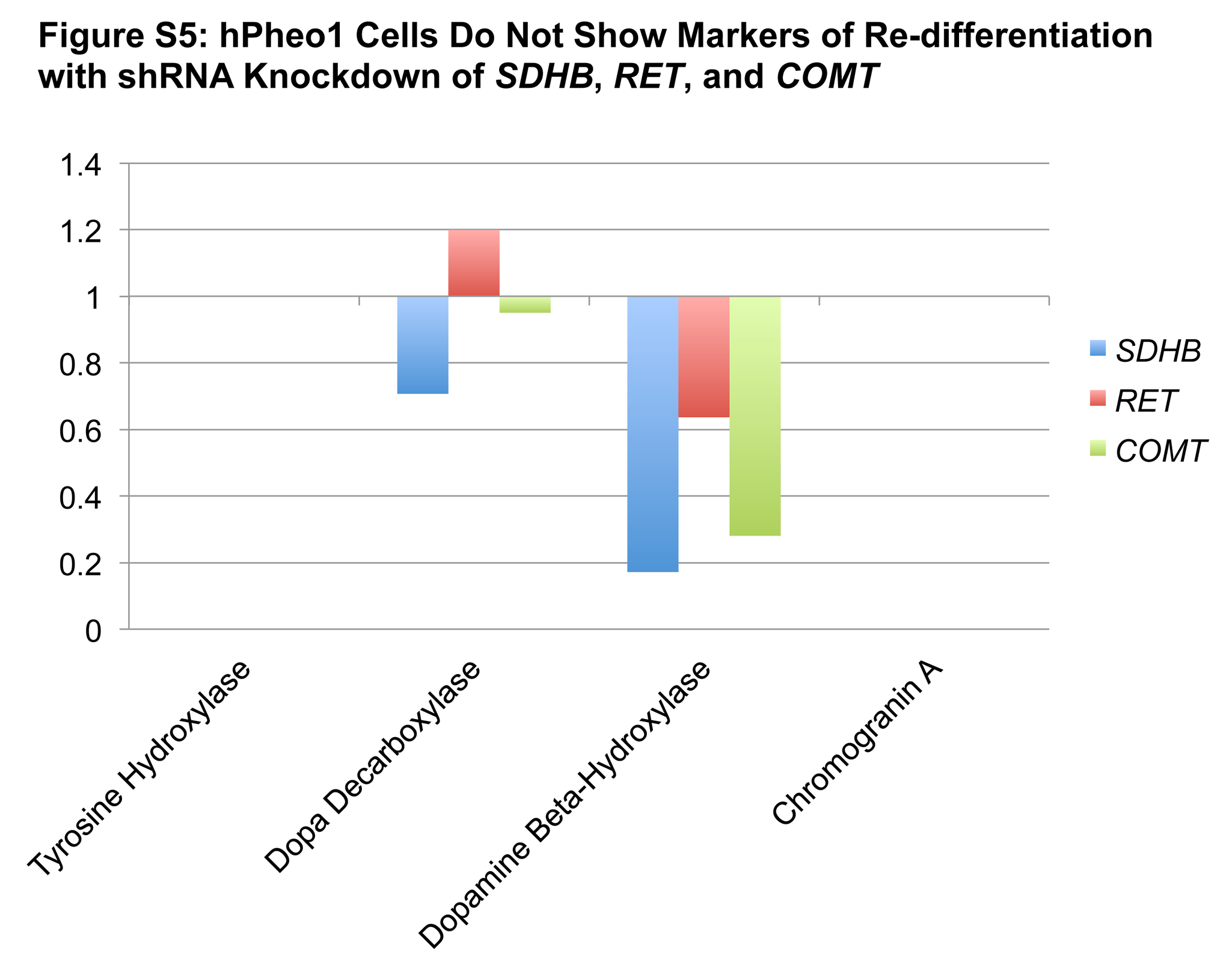

Supplement: Figure S5 — hPheo1 cells do not show re-differentiation with shRNA knockdown of SDHB , RET , and COMT when assessing changes in tyrosine hydroxylase, dopa decarboxylase, dopamine beta-hydroxylase, and chromogranin A. (TIF) [file pone.0065624.s005.tif]
